# Immunomodulation therapy with lenalidomide in follicular, transformed and diffuse large B cell lymphoma: current data on safety and efficacy

**DOI:** 10.1186/1756-8722-6-55

**Published:** 2013-08-02

**Authors:** Madhav Desai, Kate J Newberry, Jorge Romaguera, Liang Zhang, Zhishuo Ou, Michael Wang

**Affiliations:** 1 Department of Lymphoma and Myeloma, The University of Texas MD Anderson Cancer Center, 1515 Holcombe Blvd, Houston, TX 77030, Houston, TX, USA; 2 Department of Stem Cell Transplantation and Cellular Therapy, The University of Texas MD Anderson Cancer Center, Houston, TX, USA

**Keywords:** Lenalidomide, Immunomodulatory, B-cell, Lymphoma

## Abstract

Lenalidomide is an immunomodulatory agent which has been approved for multiple myeloma. Lenalidomide is also effective in and tolerated well by patients with follicular lymphoma, diffuse large B-cell lymphoma, and transformed large cell lymphoma. This review summarizes the results of current preclinical and clinical studies of lenalidomide, alone or in combination with the monoclonal antibody rituximab, as a therapeutic option for these three lymphoma types. This review will serve as a tool guiding future clinical investigations to improve survival rates for these three lymphomas.

## Introduction

Originally used to treat morning sickness and aid sleep, thalidomide was banned in the United States in the 1960s because it was linked to birth defects. But in 1999, it was found to be effective in managing relapsed or refractory multiple myeloma (MM) [1]. In 2006, after thalidomide was shown to be effective in a large, multicenter phase III trial, [2] the U.S. Food and Drug Administration granted accelerated approval for its use in newly diagnosed MM. But severe side effects including neuropathy, thromboembolism, somnolence and fatigue, as well as the emergence of resistance have limited its use [3,4]. Attempts to find better and safer alternatives to thalidomide have resulted in the development of a novel class of structural analogues called immunomodulators [5,6]. Immunomodulators, such as lenalidomide and pomalidomide, possess a mechanism of action similar to that of thalidomide but with more potent immune enhancing actions [5]. Compared with thalidomide, lenalidomide possesses an additional amino (NH_2_) group at position 4 of the phthaloyl ring and loss of the carbonyl (C = O) group at position 2.

Lenalidomide has also shown activity in non-Hodgkin lymphoma (NHL) in pivotal phase II clinical trials [7-9]. However, because these trials included many lymphoma subtypes without sufficient statistical power for each subtype, the activity of lenalidomide in specific NHL subtypes was not well defined. To date, there has been no collective review on lenalidomide in follicular lymphoma (FL), diffuse large B-cell lymphoma (DLBCL), or transformed large cell lymphoma (TmL). Options are limited for these aggressive NHLs and novel agents are needed to improve survival times. Here, we review the results of recent preclinical and clinical studies of lenalidomide in FL, DLBCL, and TmL, and highlight potential insights to apply to future clinical trials.

### Preclinical experience with lenalidomide

**Figure 1:**
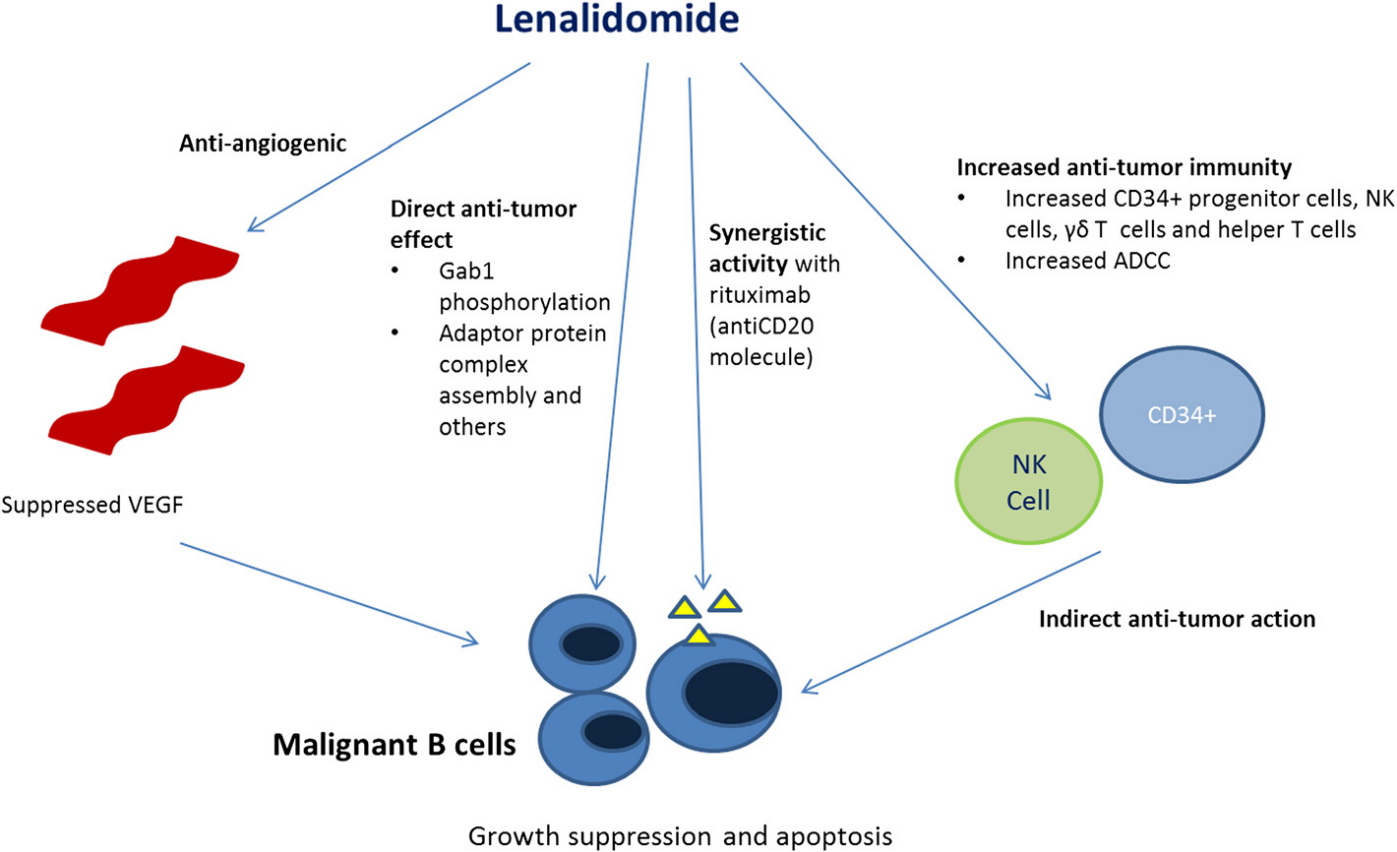
**Lenalidomide possesses a diverse set of mechanism of action in the tumor microenvironment.** These actions include direct growth inhibition, apoptosis induction, anti-tumor immune response, and anti-angiogenesis.

The precise mechanism of action of lenalidomide is not known [10]. It inhibits tumor growth, induces apoptosis, and directly kills tumor cells in B-cell NHL cell lines [11,12]. Lenalidomide also increases peripheral blood mononuclear cell activity, which can cause tumor cell apoptosis [12]. It also enhances antibody-dependent cell-mediated cytotoxicity (ADCC) and could partially overcome rituximab resistance in rituximab-treated NHL cell lines [13,14]. In addition, lenalidomide has significant anti-angiogenic effects, including interactions with the tumor cell microenvironment, endothelial cells, and vascular endothelial growth factor [15,16]. Lenalidomide can effectively repair the immunological synapse dysfunction induced by FL cells, thereby surpassing the immune-evasion mechanisms of tumor cells [17]. Taken together, lenalidomide possesses a diverse set of mechanism of action in the tumor microenvironment (Figure 1).

Single-agent lenalidomide has been shown to be active in B-cell lymphoma, [11,12] but data from preclinical studies of DLBCL and FL are limited. Table 1 summarizes the results from preclinical studies of lenalidomide in DLBCL and FL cell lines. To date, there are no published preclinical studies that have evaluated the activity of lenalidomide specifically in patients with TmL.

**Table 1:** Preclinical studies with single-agent lenalidomide in NHL

**Study**	**Cell line**	**Study findings**
Gandhi et al. [11]	Namalwa CSN.70 (Burkitt lymphoma) cells	• Len inhibited proliferation of B-cell lymphoma cells and interfered with Gab1 phosphorylation and adaptor protein complex assembly.
Reddy et al. [14]	Rtx-resistant cell lines created from Raji (Burkitt lymphoma) cells	• Len improved Rtx anti-tumor activity and partially overcame Rtx resistance by augmenting ADCC.
Verhelle et al. [50]	Namalwa (Burkitt lymphoma) and normal CD34+ progenitor cells	• Len plus pomalidomide inhibited proliferation of malignant B cells while expanding population of normal CD34+ progenitor cells.
Zhu et al. [12]	Raji (Burkitt lymphoma), K562 (CLL), PC-3 (prostate cancer), and PBMC cells	• Len plus pomalidomide induced apoptosis through NK cell activation.
Gaidarova et al. [51]	MCL and PBMC (healthy donor) cells	• Len enhanced anti-tumor effects of γδ T-cells against MCL.
Zhang LH et al. [52]	MCL, DLBCL, and FL cells	• Len induced direct anti-proliferative effects against each NHL subtype.
		• Len inhibited high vascular endothelial growth factor levels seen in cell lines.
		• These effects were associated with increased expression of tumor suppressor proteins p21 and SPARC.
Escoubet-Losachet al. [53]	Namalwa (Burkitt lymphoma) and LP-1 (MM) cells	• Len plus pomalidomide induced p21 WAF-1 expression in lymphoma and MM through an LSD1-mediated epigenetic mechanism.
Ramsay et al. [17]	FL, DLBCL, and TmL cells (fresh samples)	• FL cells induced T-cell immunological synapse dysfunction that were repaired with len.

Rtx = rituximab, CLL = chronic lymphocytic leukemia, PBMC = peripheral blood mononuclear cells, MCL = mantle cell lymphoma, FL = follicular lymphoma, MM = multiple myeloma, TmL = transformed lymphoma, DLBCL = diffuse large B cell lymphoma, NHL = non-hodgkin lymphoma, ADCC = antibody-dependent cell-mediated cytotoxicity.

Although the mechanism of action of lenalidomide has been extensively studied, its precise molecular target was undetermined until recently. In 2010, researchers discovered that a cellular protein called cereblon (CRBN) is associated with thalidomide’s teratogenicity and that CRBN is also required for the anti-tumor effects of lenalidomide and similar drugs, as well as the development of resistance to this class of drugs [18,19]. When CRBN is depleted, gene expression changes induced by the drug are suppressed. Future trials should focus on the possible role of CRBN as a biomarker for clinical assessment of lenalidomide efficacy.

Rituximab is a monoclonal antibody against CD20 and has shown activity in B-cell malignancies, which led to its approval for treatment of many CD20+ B-cell NHL [20]. Studies of B-cell lymphomas have shown that the combination of lenalidomide and rituximab is indeed effective and that it is a rational synergistic combination (Table 2) [13,21-24]. In 2005, Hernandez-Ilizaliturri et al. reported that the lenalidomide-rituximab combination inhibited cell growth and induced apoptosis in NHL cell lines, including DLBCL cell lines (SU-DHL-4 and SU-DHL-10), more than either agent alone [22]. This and other studies have shown that lenalidomide increases NK cell function and population, enhances NK cell- and monocyte-mediated ADCC, alters the production of cytokines by dendritic cells, and inhibits angiogenesis, which all serve to increase the anti-tumor effects of rituximab [13,22,23]. The lenalidomide-rituximab combination has also shown synergistic effects in most of the NHL cell lines tested. The lenalidomide-rituximab combination was shown to reduce tumor burden and increase survival in B-cell lymphoma (NHL)-bearing SCID mice [21,22].

**Table 2:** Preclinical studies with the combination of lenalidomide and rituximab in NHL

**Study**	**Cell line**	**In vivo model**	**Study findings**
Hernandez-Ilizaliturri et al. [22]	Raji (Burkitt lymphoma) and SU-DHL-4 and SU-DHL-10 (DLBCL) cells	SCID mouse (Burkitt Lymphoma)	• Len augmented NK cell function and increased anti-tumor effects of Rtx against B-cell lymphomas.
			• Len-Rtx enhanced anti-tumor activity in SCID-mouse lymphoma model.
Wu et al. [13]	Raji and Namalwa (Burkitt lymphoma), Farage (DLBCL), Jeko-1 (MCL), and primary B-CLL cells	-	• Len enhanced NK cell- and monocyte-mediated ADCC of Rtx-treated CD20+ tumor cells.
			• Len has strong potential to enhance Rtx-mediated killing of NHL cell lines.
Reddy et al. [23]	Raji (Burkitt lymphoma) cells	SCID mouse (Burkitt Lymphoma)	• Len-Rtx enhanced anti-tumor effects.
			• These effects were caused by modulation of the immune system mediated by dendritic cells and NK cells, which changed the cytokine milieu, and by their anti-angiogenic effects.
Gaidarova et al. [54]	Jeko-1 (MCL) cells	-	• Len-Rtx treatment of MCL cells enhanced NK cell-mediated synapse formation and cell killing.
Gandhi et al. [55]	DoHH-2 (FL), Rec-1 (MCL), Farage (DLBCL), and fresh FL cells		• Len-Rtx induced anti-proliferative and anti-apoptotic effects in FL cells in vitro and in vivo through Bcl-2 activation.
Zhang et al. [21]	SP53, MINO, Grant 519 cells (MCL) and fresh patient samples (MCL)	SCID mouse (MCL)	• Len-Rtx had a synergistic therapeutic effect on MCL cells by enhancing apoptosis and Rtx-dependent NK cell-mediated cytotoxicity.
			• Len-Rtx decreased tumor burden and prolonged survival of MCL-bearing SCID mice.
Gaidarova et al. [56]	Jeko-1 (MCL) and B-CLL cells	-	• Len induced capping of CD20 and cytoskeletal proteins to enhance Rtx immune recognition of malignant B-cells.

Len = lenalidomide, Rtx = rituximab, MCL = mantle cell lymphoma, FL = follicular lymphoma, DLBCL = diffuse large B-cell lymphoma, CLL = chronic lymphocytic leukemia, NK cells = Natural Killer cells, ADCC = antibody-dependent cell-mediated cytotoxicity.

### Follicular lymphoma

FL is the most common indolent NHL [25,26]. The World Health Organization grading system categorizes FL into the following grades on the basis of morphologic features, which correlate with the clinical course [27]. FLG1/2 is indolent in nature, while FLG3 [grade 3A and 3B] has a more aggressive course similar to DLBCL [28]. The majority of patients present with advanced-stage disease and are not curable with standard chemotherapy ultimately leading to disease relapse. FL has been characterized by repeated responsiveness to treatment, but the response is mostly partial and of short duration. Relapse or transformation leads to death in most cases.

Promising results from preclinical studies and clinical success with MM led investigators to design clinical trials with lenalidomide as a single agent against NHL. Three large phase II multi-center clinical trials, NHL-001, NHL-002, NHL-003, studied the activity and tolerability of single-agent lenalidomide in relapsed or refractory B-cell NHL. In all three clinical trials, oral lenalidomide at a dose of 25 mg was self-administered for days 1–21 of each 28-day cycle. These trials stratified the response data by NHL subtype and response rates are presented in Table 3.

**Table 3:** Clinical trials with single-agent lenalidomide (25 mg, days 1–21) against FL, DLBCL, and TmL

**Study**	**No. of patients**	**ORR**	**CR/uCR**	**PR**	**MDR**^**ǂ**^	**PFS**^**ǂ**^	**OS**^**ǂ**^	**F/U**^**ǂ**^	**Most common**
									adverse events (grade 3 & 4)*
Untreated FLG1/2									
Single agent lenalidomide has not been studied for initial therapy of FLG1/2.									
Relapsed or refractory FLG1/2									
NHL-001:	22	27	9	18	>16.5*	4.4*	N/A	4.4*	Neutropenia (30% & 16%)
Witzig et al. [9]									
									Thrombocytopenia (14% & 5%)
Untreated FLG3									
Single agent lenalidomide has not been studied for initial therapy of FLG3.									
Relapsed or refractory FLG3									
NHL-002:	5	60	0/20	40	6.2*	4*	N/A	3.7*	Neutropenia (24.5% & 8.2%)
Wiernik et al. [7]									
									Thrombocytopenia (12.2% & 8.2%)
NHL-003	19	42	11	32	NR	8.9	N/A	9.2*	Neutropenia (41%)
Witzig et al. [8]									
									Thrombocytopenia (19%)
Untreated DLBCL									
Single agent lenalidomide has not been studied for initial therapy of DLBCL.									
Relapsed or refractory DLBCL									
NHL-002:	26	19	4/8	8	6.2*	4*	N/A	3.7*	Neutropenia (24.5% & 8.2%)
Wiernik et al. [7]									
									Thrombocytopenia (12.2% & 8.2%)
NHL-003	108	28	7	20	4.6	2.7	N/A	9.2*	Neutropenia (41%)
Witzig et al. [8]									Thrombocytopenia (19%)
Untreated TmL									
Single agent lenalidomide has not been studied for initial therapy of TmL.									
Relapsed or refractory TmL									
NHL-002:	3	33	0	33	6.2*	4*	N/A	3.7*	Neutropenia (24.5% & 8.2%)
Wiernik et al. [7]									
									Thrombocytopenia (12.2% & 8.2%)
NHL-003:	33	45	21	24	12.8	5.4	N/A	9.2*	Neutropenia (41%)
Witzig et al. [8]									Thrombocytopenia (19%)

*Value is for whole study group.

ǂMedian values are shown in months.

N/A = data not available, OS = overall survival, PFS = progression free survival, DR = duration of response, FL = follicular lymphoma, TmL = transformed lymphoma, ORR = overall response rate, CR = complete response, uCR = unconfirmed CR, PR = partial response, F/U = follow-up period.

NHL-001 was a phase II, single-arm, multicenter study which enrolled 43 patients with indolent NHL, including 22 patients with FLG1/2 [9]. FLG1/2 patients had received a median of 3 (range, 1–17) prior systemic therapies. An ORR of 27% was observed, while 9% of patients achieved CR. The median progression-free survival time (PFS) for the FLG1/2 group was not reported, but the median PFS time for all 43 patients enrolled in the study was 4.4 months (range, 2.5-10.4 months). The most common grades 3 and 4 hematological toxicities were neutropenia (30% and 16%) and thrombocytopenia (14% and 5%).

NHL-002 was a phase II single-arm study conducted at multiple centers in the U.S. and enrolled 49 patients with relapsed or refractory aggressive NHL (FLG3, DLBCL, TmL, and mantle cell lymphoma [MCL]). Patients had received a median of 4 (range, 1 to ≥ 5) prior treatment regimens [7]. The ORR for all patients was 35%; the partial response (PR) rate was 22% and the CR/unconfirmed CR (CRu) rate was 12%.%). In the NHL-002 trial, three (60%) of the five patients with relapsed and/or refractory FLG3 achieved a response, one (20%) of which was an unconfirmed CR [7]. The median PFS duration was 4 months (range, 0–14.5 months), and the median follow-up time was 3.7 months (range, 0–12.8 months). Grade 4 adverse events were neutropenia (8.2%) and thrombocytopenia (8.2%). The most common grade 3 adverse events were neutropenia (24.5%), leukopenia (14.3%), and thrombocytopenia (12.2%).

After the initial promising data from these small trials, a larger international phase II, single-arm, open-label study of lenalidomide in 217 patients with biopsy-proven relapsed or refractory aggressive DLBCL, FLG3, TmL, or MCL was initiated (NHL-003). In the NHL-003 trial, 217 patients had received a median of 3 (range, 1–13) prior treatment regimens [8]. The ORR was 35%; the PR rate was 22% and the CR/CRu rate was 13%. The median PFS was 3.7 months, and the median follow-up period was 9.2 months. The most common grade 3 or 4 adverse events were neutropenia (41.0%), thrombocytopenia (19.0%), and anemia (9.2%). In the NHL-003 trial, eight of the 19 patients with FLG3 (42%) achieved a response, two (11%) of whom achieved CR; the median PFS duration in this trial was 8.9 months.^9^

Despite preclinical evidence of the synergistic activity and enhanced anti-tumor efficacy of the lenalidomide–rituximab combination, clinical studies testing this regimen in FL, DLBCL and TmL are limited. Published clinical data on this novel combination in patients with FL, DLBCL, or TmL are summarized in Table 4 and discussed below.

**Table 4:** Clinical trials with the combination of lenalidomide and rituximab against FL, DLBCL, and TmL

**Study**	**Regimen**	**Pt**	**ORR (%)**	**CR/uCR (%)**	**PR (%)**	**Median DR**	**Median PFS**	**Median OS**	**Median F/U**	**Most common**
										adverse events (grade 3 & 4)*
Untreated FL (not stratified)										
Fowler et al. [29,30]	Len: 20 mg, D1-21	46	98	87	11	N/A	2-yr PFS* 89%	N/A	22 months	Neutropenia (40%)
	Rtx: 375 mg/m^2^, D1									Thrombocytopenia (4%)
Relapsed or refractory FL (not stratified)										
Dutia et al. [32]	Len: 20 mg, D1-21	16	86	50	36	N/A	13 months*	N/A	9 months*	Lymphopenia (25%)
	Rtx: 375 mg/m^2^, D15 of C1, 1/wk × 4									Neutropenia (19%)
										Hyponatremia (19%)
Wang et al. [34] [only FLG3]	Len: 20 mg, D1-21	4	25	0	25	10.2 months*	2.0 months	25.6 months	24.6 months*	Neutropenia (31% & 22%)
	Rtx: 375 mg/m^2^,									Thrombocytopenia (18% and 16%)
	1/wk × 4, only C1									
Leonard et al. [33]	Len: 15 mg C1;	45	49	13	36	N/A	EFS 1.2 years	N/A	1.5 years	Neutropenia (16%)
	20 mg C2-12, D1-21									Thrombosis (16%)
										Fatigue (9%)
	Len: 15 mg C1;	44	75	32	43	N/A	EFS 2.0 years	N/A	1.5 years	Neutropenia (19%)
	20 mg C2-12, D1-21									Fatigue (14%)
	Rtx: 375 mg/m^2,^, 1/wk × 4									Thrombosis (4%)
Untreated DLBCL										
Lenalidomide plus rituximab combination as a front-line therapy has not been studied for untreated DLBCL.										
Relapsed or refractory DLBCL										
Zinzani et al. [41]	Len: 20 mg, D1-21,	23	35	35	0	N/A	1-yr DFS 34.8%	18-month OS 55%	16 months	Neutropenia (30%)
	Rtx: 375 mg/m^2^, D1-21 (during induction)									Thrombocytopenia (14%)
Wang et al. [34]	Len: 20 mg, D1-21	32	28	22	6	10.2 months*	2.8 months	10.2 months	24.6 months*	Neutropenia (31% & 22%)
	Rtx: 375 mg/m^2^;									Thrombocytopenia (18% and 16%)
	1/wk × 4, only C1									
Untreated TmL										
Lenalidomide plus rituximab combination as a front-line therapy has not been studied for untreated TmL.										
Relapsed or refractory TmL										
Wang et al. [34]	Len: 20 mg, D1-21	9	56	33	22	10.2 months*	4.3 months	11.5 months	24.6 months*	Neutropenia (31% & 22%)
	Rtx: 375 mg/m^2^.									
	1/wk × 4, only C1									Thrombocytopenia (18% and 16%)

*Value is for whole study group.

D = day, C = cycle, FL = follicular lymphoma, N/A = data not available, NHL = non-Hodgkin lymphoma, R/R = relapsed or refractory, Len = lenalidomide, Rtx = rituximab, EFS = event-free survival, OS = overall survival, PFS = progression-free survival, DR = duration of response, FL = follicular lymphoma, TmL = transformed lymphoma, CR = complete response, uCR = unconfirmed CR, PR = partial response, ORR = overall response rate, F/U = follow-up period.

Fowler et al. recently reported promising efficacy and tolerability data with lenalidomide plus rituximab in untreated indolent NHL from a single-center phase II trial [29]. Patients were not stratified for World Health Organization grading of FL. Forty-six patients with previously untreated, advanced stage FL had a high ORR of 96% and a CR rate of 87%. The PFS rate at the end of 2 years was 89% after a median follow up of 22 months. There was a low incidence of adverse events; the most significant grade 3 or 4 event was neutropenia (40%) [30]. Thrombocytopenia was seen in 4% of patients. The most common grade 3/4 non-hematological adverse events were: rash (7%) and muscle pain (6%). These findings indicate a highly promising role for the lenalidomide-rituximab combination as a front-line regimen against FL. Immunophenotyping and gene expression profiling (GEP) of peripheral blood samples from FL patients at baseline and end of cycle 6 from this study showed that lenalidomide can induce multiple changes in the immune system including down-regulation of certain genes mediating B cell migration and proliferation [31].

Dutia et al. were the first to report promising activity of the lenalidomide-rituximab combination for relapsed or refractory FL [32]. For the 16 patients in that study the ORR was 86%, and 8/16 had CR. The median PFS duration was 13 months. Lymphopenia (25%), neutropenia (19%) and hyponatremia (19%) were significant grade 3 or 4 adverse events. Stratified responses for FL subtypes are not available. Leonard et al. directly compared single-agent lenalidomide to the lenalidomide-rituximab combination in recurrent FL in a phase II randomized controlled trial (CALGB 50401) [33]. In both treatment arms, patients were treated with 15 mg of lenalidomide in cycle 1 (on days 1–21 of a 28-day cycle) followed by 20 mg of lenalidomide during cycles 2–12. In the lenalidomide-only arm, which consisted of 45 patients, the ORR was 49% with an event-free survival duration of 1.2 years. Significant grade 3 or 4 toxicities were neutropenia (16%) thrombosis (16%), and fatigue (9%). In the lenalidomide-rituximab arm, which consisted of 44 patients, the addition of 375 mg/m^2^ rituximab once weekly for 4 weeks increased the ORR to 75% and the event-free survival time to 2 years. Slightly more grade 3 or 4 neutropenia (19%) and fatigue (14%) was observed but less thrombosis (4%) than in the lenalidomide-only arm [33]. Thus, compared with lenalidomide alone, the lenalidomide-rituximab combination was more effective without significantly more toxicity against relapsed or refractory FL.

Highly promising activity has been reported in untreated, advanced FL as well as in recurrent, relapsed FL, but because FL patients were not stratified by WHO grading in these trials, it is not possible to determine whether there are differences in the activity of lenalidomide-rituximab between those with indolent (FLG1/2) and aggressive disease (FLG3) [30,33]. Our group recently published the results of a single-center phase II trial using lenalidomide plus rituximab for 45 NHL patients, including four with FLG3 [34]. In that trial, only one patient with FLG3 achieved a partial response (25%). The median overall survival duration was 25.6 months, which was the highest among all the NHL subtypes in this study [34]. Because lenalidomide alone can elicit an ORR of 42-60% in patients with relapsed or refractory FLG3 [7,8], the addition of the monoclonal antibody rituximab might have been expected to elicit a higher response rate; however, given the small sample size it is hard to draw any conclusions. Because FLG3 is a rare lymphoma, multi-center international trials will be necessary to gauge the actual activity of this combination in these patients.

### Diffuse large b-cell lymphoma

DLBCL is the most common subtype of NHL [25,26]. It is divided into three major categories based on gene expression signatures: activated B-cell-like (ABC), germinal center B-cell-like (GCB), and primary mediastinal B-cell lymphoma (PMBL) [35,36]. The genetic differences might be responsible for their different rates of response to chemotherapy. More than one-third of patients relapse or become refractory to standard chemotherapy [37]. Even with the best second-line agents, the 3-year EFS remains low (21%) [38]. Relapse or progressive disease (refractory) is a major cause of death in DLBCL patients. In brief, novel agents are sorely needed for this NHL subtype.

Single-agent lenalidomide has shown activity in clinical trials to. There were 26 patients with relapsed or refractory DLBCL in the NHL-002 trial. The ORR was 19% and the CR rate was 4% (uCR 8%) [7]. In the NHL-003 trial, which enrolled 108 patients with relapsed or refractory DLBCL, the ORR was 28% and the CR rate was 8% [8]. In the latter trial, the median PFS for DLBCL patients was 2.7 months and the median response duration was 4.6 months.

There is a lack of conclusive data regarding the clinical response of DLBCL subtypes to lenalidomide as a single agent or in combination with chemotherapy. Hernandez-Ilizaliturri et al. investigated the activity of single-agent lenalidomide against relapsed or refractory DLBCL, which was stratified into GCB-like (n = 23) and non-GCB-like subtypes (n = 17) according to the Hans algorithm [39]. In that study, lenalidomide was more effective in treating non-GCB-like DLBCL (mainly the activated B-cell-like variant) than GCB-like DLBCL (ORR, 52.9% vs. 8.7%). Recently, Zhang et al. found that lenalidomide has direct antitumor activity against DLBCL cells, which is mediated by blocking IRF-4 expression and the BCR-NF-kB signaling pathway in a cereblon dependent manner [40]. Lenalidomide was found to preferentially suppress proliferation of ABC-DLBCL cells in vitro and delay tumor growth in a human tumor xenograft model with minimal effect on non-ABC-DLBCL cells. In order to more accurately define DLBCL subtypes and those patients who respond to lenalidomide, future trials enrolling DLBCL patients should stratify them on the basis of major subtypes based on gene signatures to assess whether cell of origin (COO) and clinical response are associated. This will allow us to identify the population that can derive the most benefit from lenalidomide.

Although the lenalidomide-rituximab combination has shown considerable activity in preclinical studies, to date, only 2 clinical studies have tested this combination in relapsed or refractory DLBCL. Zinzani et al. reported the results of a phase II clinical trial testing the combination in a group of 23 elderly patients (≥ 65 years) with relapsed or refractory DLBCL [41]. Patients were treated with oral lenalidomide (20 mg/day for 21 days of each 28-day cycle) and rituximab (375 mg/m^2^ on days 1 and 21 of each 28-day cycle) for 4 cycles followed by a maintenance phase with lenalidomide only for another 8 months. The median number of prior therapies was 3 (range, 2–8). A CR was achieved in 8 patients (35%) at the end of the maintenance phase. The 1-year disease-free survival (DFS) rate was 35%, and the 18-month overall survival rate was 55%. The most common grade 3 or 4 adverse events were neutropenia (30%) and thrombocytopenia (14%) [41].

Our recently completed trial included 32 patients with relapsed or refractory DLBCL [34]. The patients received 20 mg of oral lenalidomide on days 1–21 of each 28-daycycle and intravenous rituximab (375 mg/m^2^) for 4 weekly doses during the first cycle. The median number of prior lines of therapy was 3 (range, 1–4), and the median follow-up time was 24.6 months (range, 12.4-43.7 months). The ORR was 28%, and the CR rate was 22%. The median overall survival time was 10.2 months. The most common grade 3–4 hematological toxicities for the whole study population were neutropenia (53%) and thrombocytopenia (34%).

Compared with the clinical data for single-agent lenalidomide in relapsed or refractory DLBCL, for which 4-8% patients achieved CR, [7,8] these two clinical studies using the lenalidomide-rituximab combination reported a substantially higher proportion of patients achieving a CR (28-35%) [34,41]. The findings are quite promising, and the lenalidomide-rituximab combination should be studied further in a larger population of patients with relapsed or refractory DLBCL.

Lenalidomide as a single agent as well as in combination with rituximab can be a good alternative as a maintenance regimen once CR is achieved with high dose chemotherapy or following ASCT. One such study demonstrated lenalidomide as a maintenance therapy has promising clinical activity following standard chemotherapy R-CHOP and resulted in superior survival outcomes in DLBCL patients with high risk prognostic features (IPI ≥ 3). The 2-year PFS and DFS were 92% and 100%, respectively, for those who received lenalidomide maintenance (25 mg/day for 21 days of 28-cycle) for one year [42]. Survival rates were slightly lower with the lenalidomide-rituximab combination than with single agent lenalidomide. Larger studies comparing maintenance regimens may shed light on this disparity; still, it is enough to conclude that lenalidomide is a good tolerable strategy for keeping patients relapse-free once CR is achieved.

Nowakowski et al. tested adding lenalidomide to conventional R-CHOP chemotherapy for frontline treatment of aggressive B-cell lymphomas, including DLBCL and FLG3. The phase I study demonstrated that 25 mg lenalidomide give on days 1–10 of the cycle can be combined with R-CHOP without the addition of significant toxicity [43]. A phase II study showed that addition of lenalidomide to R-CHOP is an effective and safe option for initial therapy of patients with DLBCL and FLG3 [44]. There were 47 patients with newly diagnosed DLBCL and 4 with newly diagnosed FLG3. The ORR and CR rates were 98% and 83%. The PFS was 73% at the end of 1-year. The most common hematological toxicities were grade 3 and 4 thrombocytopenia (20% and 20% of patients, respectively) and grade 3 and 4 neutropenia (18% and 71% of patients, respectively). This study provided pivotal evidence for further studying the combination of R-CHOP and lenalidomide for newly diagnosed aggressive B-cell lymphomas.

### Transformed large cell lymphoma

When an indolent lymphoma turns into an aggressive, rapidly progressive lymphoma both clinically and microscopically, it is defined as a TmL [45]. Any indolent B- or T-cell lymphoma can transform, but FL is the most likely to [46,47]. Indolent FL can undergo transformation to become DLBCL, Burkitt lymphoma, or (rarely) lymphoblastoid lymphoma. The risk of FL transformation is 3% per year after initial diagnosis with a very short post-transformation survival (1.7-2.7 years) [45,48]. Management of TmL is challenging and there is no standard approach for treatment.

Because TmL is a rare form of lymphoma, clinical data on the activity of lenalidomide are limited. Three patients were enrolled in the NHL-002 study, 1 of whom (33%) achieved a PR [7]. The NHL-003 trial included 33 TmL patients, with 15 (45%) achieving a response, and among those, 7 (21%) achieved a CR [8]. The median PFS was 5.4 months, and the median follow-up time was 9.2 months. In a separate sub-analysis of the 33 patients with TmL in the NHL-003 study, Czuczman et al. found lenalidomide was effective for patients with transformed FL (ORR, 56.5%) but not those with transformed chronic or small lymphocytic lymphoma (ORR, 0%) [49]. Larger trials with stratified histological subtype analyses are required to validate those findings.

To date, there has been limited experience with the use of the lenalidomide-rituximab combination in TmL. Since single-agent lenalidomide has been shown to be effective against TmL^8,9^ and rituximab [20] has been shown to be effective against all aggressive NHLs studied to date, this combination may well be effective against TmL. Our recent study included nine patients with previously treated TmL [34]. The ORR was 56%, and the CR rate was 33% with a median OS of 11.5 months. These findings are promising but further investigation in a large-scale, multicenter, international trial of the lenalidomide-rituximab combination in patients with relapsed or refractory TmL is necessary.

## Conclusion

In conclusion, novel agents with enhanced efficacy and fewer side effects are needed for patients with relapsed and/or refractory DLBCL, FL and TmL, the most common of NHL subtypes. Lenalidomide is a good candidate agent due to its unique immunomodulatory properties, ability to prolong patient survival, and good safety profile. Many clinical trials have been designed and are enrolling patients to assess the activity and safety of frontline, maintenance, or salvage lenalidomide —alone, with rituximab, or with other agents — against FL, DLBCL, and TmL. Randomized controlled clinical trials of lenalidomide as a single agent and in combination with rituximab and other targeted therapies should be conducted with larger populations stratified by disease subtype and gene expression profiles to confirm the initial findings and to move lenalidomide into the growing arsenal of frontline therapies for aggressive NHLs.

## Competing interests

The authors declare that they have no competing interests.

## Authors’ contributions

MD and MW conceptualized, drafted, edited, proofread and reviewed the manuscript. JR, KN, LZ and ZO gave suggestions, proofread and reviewed the work. All authors read and approved the final manuscript.
